# Body composition and cardiorespiratory fitness in overweight or obese people post COVID-19: A comparative study

**DOI:** 10.3389/fphys.2022.949351

**Published:** 2022-09-21

**Authors:** Maurício Medeiros Lemos, Gustavo Rocha Cavalini, Carlos Renato Pugliese Henrique, Victor Augusto Santos Perli, Glória de Moraes Marchiori, Luciana Lozza de Moraes Marchiori, Ana Flávia Sordi, Solange Marta Franzói de Moraes, Solange de Paula Ramos, Pablo Valdés-Badilla, Jorge Mota, Braulio Henrique Magnani Branco

**Affiliations:** ^1^ Interdisciplinary Laboratory of Intervention in Health Promotion, Cesumar Institute of Science, Maringá, Brazil; ^2^ Postgraduate Program in Health Promotion, Cesumar University, Paraná, Brazil; ^3^ Medicine Course, Department of Health Sciences, Cesumar University, Maringá, Brazil; ^4^ Department of Physical Education, State University of Maringá, Maringá, Brazil; ^5^ Department of Physical Education, State University of Londrina, Londrina, Brazil; ^6^ Department of Physical Activity Sciences, Faculty of Education Sciences, Universidad Católica del Maule, Talca, Chile; ^7^ Sports Coach Career, School of Education, Universidad Viña del Mar, Viña del Mar, Chile; ^8^ Research Centre of Physical Activity, Health and Leisure, Faculty of Sports; Laboratory for Integrative and Translational Research in Population Health (ITR), University of Porto, Porto, Portugal; ^9^ Physiology and Nutrition Department, Clinisport Prime, Maringa, Brazil

**Keywords:** exercise test, outcome and process assessment, health care, delivery of health care, physical fitness, COVID-19, obesity

## Abstract

The present study aimed to evaluate the body composition and cardiorespiratory fitness of overweight or obese people after COVID-19. 171 volunteers of both sexes (men, *n =* 93 and women, *n =* 78) between 19 and 65 years old were allocated into three groups according to the severity of their symptoms of COVID-19: non-hospitalized people/mild symptoms (*n =* 61), hospitalized (*n =* 58), and hospitalized in an intensive care unit-ICU (*n =* 52). Two laboratory visits were carried out 24 h apart. First, a medical consultation was carried out, with subsequent measurement of body weight and height (calculation of body mass index) and body composition assessment via electrical bioimpedance. After 24 h, a cardiorespiratory test was performed using the Bruce protocol, with a direct gas exchange analysis. Hospitalized individuals had significantly higher values for fat mass and body fat percentage than non-hospitalized individuals (*p <* 0.05). Significantly higher values were found for heart rate (HR) and peak oxygen consumption (VO_2_peak) for individuals who were not hospitalized when compared to those hospitalized in the ICU (*p <* 0.05). Significantly higher values for distance, ventilation, and the relationship between respiratory quotient were found for non-hospitalized individuals compared to hospitalized individuals and those in the ICU (*p <* 0.05). After the cardiorespiratory test, higher values for peripheral oxygen saturation (SpO_2_) were observed for non-hospitalized individuals than for all hospitalized individuals (*p <* 0.05). Diastolic blood pressure was significantly higher at the tenth and fifteenth minute post-Bruce test in hospitalized than in non-hospitalized participants (*p <* 0.05). Based on these results, proposals for cardiopulmonary rehabilitation are indispensable for hospitalized groups considering the responses of blood pressure. Monitoring HR, SpO_2,_ and blood pressure are necessary during rehabilitation to avoid possible physical complications. Volume and intensity of exercise prescription should respect the physiologic adaptation. Given lower physical conditioning among all the groups, proposals for recovering from health conditions are urgent and indispensable for COVID-19 survivors.

## Introduction

Over the past 2 years, the COVID-19 pandemic has been a cause of significant global morbidity and mortality (Hopkins, 2022). The short- and long-term impacts of severe acute respiratory syndrome coronavirus-2 (SARS-CoV-2) infection have become a challenge for promoting health recovery actions for COVID-19 survivors. Symptoms of the post-COVID syndrome (or long-term COVID) include cardiovascular, pulmonary, and neuromuscular changes, among other organic and psychosocial manifestations (Higgins et al., 2021), and persistent symptoms after medical release for the SARS-CoV-2 (Elkrief et al., 2022). These changes are also present in patients with obesity, which could potentially make them more susceptible to the development of post-COVID syndrome (Maffetone and Laursen, 2020; Higgins et al., 2021; Elkrief et al., 2022). Early diagnosis and targeted interventions for patient recovery can provide better outcomes for disease sequelae and complications of long-term post-COVID or COVID-19 syndrome (Al-Aly et al., 2021), especially in patients with risk factors for respiratory and cardiovascular alterations. However, multiple cases have presented after the least severe forms of COVID-19, with short-term (∼1 month) and long-term (≥6 months) sequelae impacting between 34.8% to 65.5% and 31.0%–67%, respectively, of COVID-19 survivors (Groff et al., 2021).

The World Health Organization (WHO) has published the “Clinical management of COVID-19: living guidance” (World Health Organization, 2021), which establishes four classifications of COVID-19 symptoms (mild, moderate, severe, and critical), which are linked to the impacts caused by SARS-CoV-2. A recent systematic review with a meta-analysis by Dessie and Zewotir (2021) pointed out that age, male sex, smoking, chronic obstructive pulmonary disease (COPD), cardiovascular disease (CVD), cancer, acute renal failure, systemic arterial hypertension, diabetes mellitus, and obesity increase the risks associated with the more severe symptoms of COVID-19. Given this, based on preventive and health promotion actions, public policies that aim to provide a healthy diet, physical activity, and tobacco control can contribute to improving the health and quality of life of the population and, consequently, reduce the costs related to the treatment of chronic diseases and the risk of developing complications from COVID-19 (Brazil, 2010).

Science has discussed better outcomes given modifiable aspects for people who contracted COVID-19, such as reduced fat mass (Maffetone and Laursen, 2020), increased cardiorespiratory fitness (Af Geijerstam et al., 2021; Brandenburg et al., 2021), increased muscle strength (Af Geijerstam et al., 2021), and a higher level of physical activity (Pitanga et al., 2021). Supporting this perspective, Sallis et al. (2021) reported that physically inactive people who had COVID-19 had a higher rate of hospitalization and ICU admission than physically active people. Pitanga et al. (2021) identified in an ecological analysis, in 26 Brazilian capitals and the federal district, an inverse correlation between the practice of physical activity in leisure time and deaths from COVID-19 (*r =* -0.44; *p =* 0.03) and lethality (*r =* −0.51; *p =* 0.01). Similarly, Barbagelata et al*.* (2021) found that asymptomatic patients had a significantly higher peak oxygen consumption (VO_2_peak) in the Bruce test than those with long-term COVID conditions. However, the authors did not control their findings by body mass index (BMI) and the severity of cases, i.e., whether there was a need for in-patient or ICU admission for treatment of COVID-19. This is a relevant factor since the need for intubation or oxygen support may be correlated with greater deficits in respiratory capacity, which are more frequent in patients with obesity (Freire et al., 2022).

Because of this, people with obesity could present intense symptoms associated with COVID-19, linked to greater inflammation, impaired immune response, worse respiratory function, and pro-coagulant profile (Stefan et al., 2020). The possible association between anthropometric body composition and cardiorespiratory parameters and the severity of the symptoms of COVID-19 (mild, moderate, and severe/critical) has not yet been verified. Therefore, the objective of the present study was to analyze the body composition and cardiorespiratory fitness of overweight or obese people according to the severity of their symptoms of COVID-19. As a hypothesis, it is believed that higher fat mass and lower cardiorespiratory fitness are related to the severity of symptoms of SARS-CoV-2 survivors.

## Materials and methods

### Experimental design

This cross-sectional, experimental, and comparative study was conducted between August and December 2021 in Maringá, Paraná, Brazil. The present study recruited people with COVID-19 who were not hospitalized (no admission), hospitalized, or admitted to the intensive care unit (ICU) via referrals from the Municipal Hospital of Maringá through TV, radio, and social media dissemination. Interested parties contacted the Interdisciplinary Laboratory for Intervention in Health Promotion (LIIPS) multidisciplinary team at Cesumar University. Participants came to the laboratory and performed two assessments at a 24-h interval. First, the participants had a consultation with an intensive care physician. Participants answered a detailed anamnesis on personal and family history, previous illnesses, symptoms of COVID-19, hospitalization (if the patient was hospitalized), and self-reported level of physical activity (means by a validated questionnaire—presented in sections above); in addition to an anthropometric and body composition assessment (protocol previously sent to the patients), as detailed in subsequent sessions. Finally, 24 h after the first assessment, the participants completed the cardiorespiratory fitness test (see the protocol below). All participants were informed about the study’s objectives and signed informed consent forms (ICF). This study followed the recommendations proposed by resolution 466/12 of the Ministry of Health of the Brazilian Government and the Declaration of Helsinki, approved by the Research Ethics Committee from Cesumar University, under number 4,546,726.

### Participants

One hundred seventy-one volunteers of both sexes participated in this study and were allocated into three experimental groups according to the clinical picture of their acute COVID-19 infection: ICU (*n =* 52), hospital (*n =* 58), and non-hospitalized (*n =* 61). More information about the volunteers is presented in Table 1. The severity of COVID-19 was classified according to the guide “Clinical management of COVID-19: living guidance” (World Health Organization, 2021). The inclusion criteria were as follows: 1) being between 19 and 65 years old; 2) being overweight or obese; 3) having a positive diagnosis for COVID-19 via qualitative molecular testing (RT–PCR); 4) having contracted COVID-19 between 03 January/2021 and 01 July/2021; 5) having received the first dose of COVID-19 vaccine; and 6) having received medical clearance for the cardiorespiratory fitness test. As exclusion criteria, the following participants were not accepted: 1) patients with debilitating neurological diseases; 2) people with limited mobility (use of a cane or wheelchair); 3) people with a body mass index below or within normal limits; 4) people without medical clearance to perform the Bruce test; and 5) non-agreement to sign the ICF.

**TABLE 1:** General characteristics of participants of both sexes by the severity of COVID-19.

Variables	ICU (*n =* 52)	Hospital (*n =* 58)	Non-hospitalized (*n =* 61)
Age	50.3 ± 11.0	48.1 ± 13.3	48.0 ± 13.0
Gender			
Male	30 (57.7%)	30 (51.7%)	33 (54.1%)
Female	22 (42.3%)	28 (48.3%)	28 (45.9%)
Medical history			
Hypertension	18 (34.6%)	13 (22.0%)	14 (23.3%)
Diabetes	11 (21.2%)	4 (6.8%)	6 (10.0%)
Dyslipidemia	15 (28.8%)	11 (18.6%)	9 (15.0%)
COPD	0 (0%)	0 (0%)	1 (1.7%)
Asthma	0 (0%)	1 (1.7%)	0 (0%)
CAD/revascularization	2 (3.8%)	0 (0%)	4 (6.7%)
Smoking			
No	40 (76.9%)	48 (82.8%)	51 (83.6%)
Past or today	12 (23.1%)	10 (17.2%)	10 (16.4%)
Length of stay			
Hospital (d)	8.0 (4.0–13.0)	8.0 (6.0–11.0)	—
Intensive care (d)	14.0 (4.7–14.0)	—	—
Total (d)	22.0 (12.0–35.0)	8.0 (6.0–11.0)	—
Type of respiratory support			
None	1 (1.9%)	11 (19.3%)	59 (96.7%)
Catheter	35 (67.3%)	40 (69.0%)	2 (3.3%)
High flow mask	33 (63.5%)	24 (41.4%)	0 (0%)
Non-invasive mechanical ventilation	33 (63.5%)	11 (19.0%)	0 (0%)
Invasive mechanical ventilation	35 (67.3%)	0 (0%)	0 (0%)
Physical activity ≥150 min/week before COVID-19	23 (44.2%)	28 (48.3%)	43 (70.5%)*

Note: data are expressed as the mean and (±) standard deviation, minimum, maximum, and relative frequency (%); *, higher values when compared to ICU, and hospitalized groups (*p <* 0.05); COPD, chronic obstructive pulmonary disease; CAD, coronary artery disease; ICU, intensive care unit; NIMV, non-invasive mechanical ventilation; IMV, invasive mechanical ventilation. Analyze One-way ANOVA.

### Clinical evaluation

Data collection was performed in the following order: 1) blood pressure measurement after 5 min of rest in a calm and quiet place, following the VIII Brazilian Guidelines on Arterial Hypertension (Barroso et al., 2020) with the evaluators’ reproducibility for measuring blood pressure was 0.99 for the intraclass coefficient (ICC); 2) heart rate (HR) mensuration and peripheral oxygen saturation (%SpO_2_), both at rest, using a finger oximeter (Alfamed^®^, model sense 10, Lagoa Santa, Minas Gerais, Brazil) positioned on the index finger; 3) height and body mass mensuration (using a stadiometer and a scale) and body composition with electrical bioimpedance (device information is presented below); 4) a questionnaire for patient identification and initial screening regarding lifestyle, clinical history [history of surgeries, noncommunicable chronic diseases, continuous use medications and physical activity, means by the Short International Physical Activity Questionnaire/IPAQ—version validated in Brazil by Matsudo et al. (2001)—with retrospective information referring to prior SARS-CoV-2 infection was applied] and information on the clinical picture of acute COVID-19 (main signs and symptoms presented, severity of COVID-19, as well as the possible need for ventilation invasive or noninvasive mechanics and central reported sequelae); and 5) a cardiorespiratory fitness test (with a treadmill and a direct analysis of gas exchange). The patients’ information about physical activity means by IPAQ was collected before the contraction of COVID-19 for all patients, i.e., there was a retrospective completion considering the period before COVID-19 infection (there were no more collections after the COVID-19 discharge).

Medical consultation, anthropometry, and body composition assessment were performed on the first visit to the Interdisciplinary Laboratory for Intervention in Health Promotion. On the second visit, 24 h later, a cardiorespiratory fitness test was conducted using the Bruce protocol according to the Brazilian Society of Cardiology (Barroso et al., 2020).

### Anthropometry and body composition

The participants’ height was measured using a Sanny^®^ stadiometer measuring 2.20 m with a precision of 0.1 cm (Standard model, ES 2030, São Bernardo do Campo, São Paulo, Brazil); body mass was measured on a mechanical scale (Welmy^®^ mechanics with a capacity of 300 kg and precision of 100 g, Model 104A, Santa Bárbara do Oeste, São Paulo, Brazil), according to the protocol established by Freitas Junior (2018), and the body mass index (BMI) was subsequently calculated. Body composition assessment was performed using a tetrapolar electrical bioimpedance (BIA) (InBody 570^®^, Biospace Co. Ltd., Seoul, Korea) with eight tactile points and a capacity of 250 kg with an accuracy of 100 g. The volunteers were instructed to 1) fast for 4 h; 2) not to ingest liquids, including caffeine and water; 3) to abstain from the use of alcoholic beverages 2 days before the evaluations; 4) to stop exercising the day before the test; 5) to urinate 30 min before the evaluation and 6) to not wear metals on the body (Branco et al., 2019). In line with previous studies, the laboratory temperature was maintained at 24°C (Heyward, 1996; de Souza Marques et al., 2021).

### Ergospirometric test

The Bruce submaximal test was used to assess the participants’ cardiopulmonary fitness (Bruce and Kusumihosmer., 1973). This protocol was chosen based on Itagi et al. (2020) for people with obesity. The Bruce protocol consisted of seven stages lasting 3 min each. The Bruce test was performed on an Inbramed treadmill (model ATL 24, Porto Alegre, Rio Grande do Sul), with progressive increases in speed and slope. The first stage started with a speed of 2.7 km/h and a slope of 10%. At the end of each stage, the speed increased progressively and nonlinearly, increasing the slope by 2%.

### Gas exchange analyses

The VO 2000^®^ metabolic gas analyzer (Medgraphics Corp., Saint Paul, United States of America) associated with the pneumotach, which connects the silicone mask to the equipment, was used to measure the lung capacity of patients based on the following parameters: expired air (VE/min), peak oxygen consumption (VO_2_peak—mL.kg^−1^. min^−1^), oxygen pulse (O_2_/HR), respiratory exchange ratio (VCO_2_/VO_2_ - RQ), HR (bpm), total distance covered (km/h) and application of the rating of perceived exertion (RPE) at the end of the test means by the Borg scale (6–20) (Borg, 1982). As suggested by the manufacturers, the equipment was automatically calibrated at the beginning of each cardiorespiratory fitness test. The intraclass correlation coefficient reported in a previous study was 0.98 (Crouter et al., 2006). The tests were conducted by a medical team consisting of an intensive care physician, a nurse, and two exercise physiologists.

### Test monitoring

The multi-professional team constantly monitored the cardiorespiratory fitness test and was attentive to extreme tiredness, %SPO_2_, and HR responses. As mentioned earlier, physiological and RPE were monitored at each stage. All participants were instructed to respond to RPE on the Borg (Borg, 1982) 6–20 scale. The cardiopulmonary fitness test was terminated on the following occasions: 1) voluntary withdrawal of the participants; 2) RPE ≥19 a.u.; 3) RQ ≥ 1.15; 4) lower limb fatigue and/or 5) physical impossibility of maintaining intensity during the test. After the end of the Bruce test, HR and %SpO_2_ were monitored minute by minute for 15 min. SBP and DBP were measured immediately after the end of the physical assessment and every 5 min during the next 15 min.

### Statistical analysis

Based on the study by Barbagelata et al. (2021), the sample size calculation in G*Power (version 3.1, University of Dusseldorf, Germany) showed that 137 volunteers would be enough for an *α* = 0.05 and a *β* = 0.80. Statistical analyses were performed using GraphPad Prism software version 8.1.0. Data are presented as the mean ± standard deviation (SD). First, data normality was tested using the skewness-kurtosis test, considering values from 2 to −2 to indicate a need to perform parametric statistical analyses. One-way analysis of variance (one-way ANOVA) was used to locate possible differences between the groups. Data with a nonparametric distribution were analyzed using the Kruskal–Wallis test. Comparisons between pre- and post-cardiorespiratory fitness tests were performed via a two-way mixed-measures ANOVA (for repeated measures). Bonferroni’s post-hoc test was used when a significant difference was found. The significance level established for all tests was *p <* 0.05. The partial eta square (η^2^) was calculated according to the classification by Richardson (2011) using the following interpretation scale: 0.0099 [*small*], 0.0588 [*moderate*], and 0.1379 [*large*]. Cohen´s *d* was also calculated using the following rating: 0.20 [*small*], 0.80 [*moderate*], and >0.80 [*large*] (Cohen, 1988).

## Results

Table 1 presents the general characteristics of the present study participants stratified by the severity of their symptoms of COVID-19.

**FIGURE 1:**
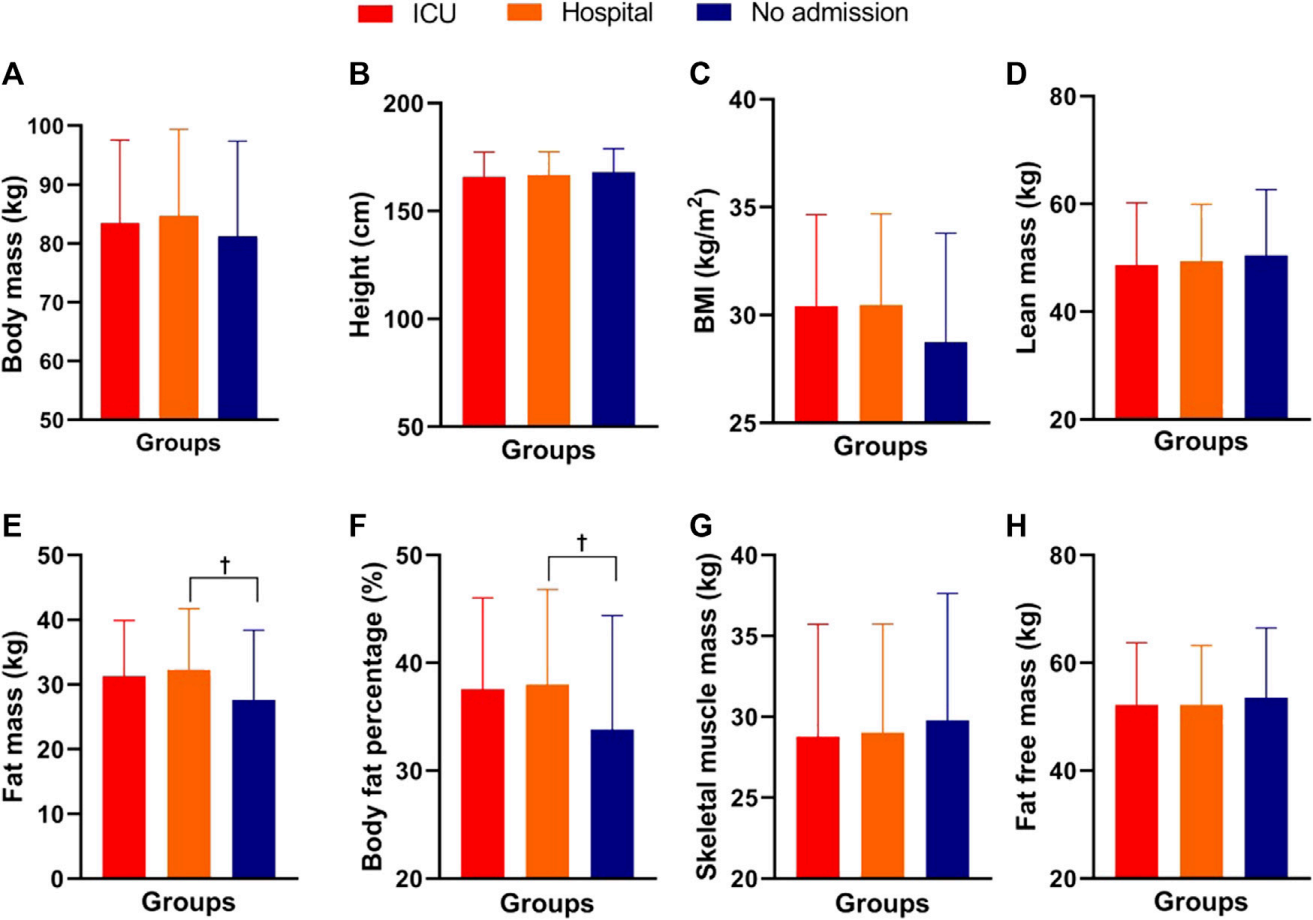
Morphological parameters of male and female COVID-19 survivors in the ICU, hospitalized, and non-hospitalized groups. Note: data are presented as the mean and (±) standard deviation; Panel **(A)** = body mass; Panel **(B)** = height; Panel **(C)** = body mass index; Panel **(D)** = lean mass; Panel **(E)** = fat mass; Panel **(F)** = body fat percentage; Panel **(G)** = musculoskeletal mass; Panel **(H)** = fat-free mass; † = COVID-19 hospitalized group had significantly higher values than the non-hospitalized COVID-19 group. One-way ANOVA with Bonferroni post hoc test. Level of significance established = *p* < 0.05.

No significant differences were observed for age, systemic arterial hypertension, dyslipidemia, or smoking among the three experimental groups (*p >* 0.05). Significant differences were detected between the groups for self-reported physical activity (F_2,168_ = 4.89; *p* = 0.008) with superior values for the non-hospitalized group when compared to ICU (*p =* 0.01 days = −0.54—*moderate*) and hospitalized (*p =* 0.04; *d* = −0.46—*moderate*). Figure 1 shows the morphological parameters of the COVID-19 survivors in the three experimental groups of the present study.

Table 2 presents physiological variables in each stage of the Bruce test, and Table 3 presents subjective variables and frequencies of participants in each stage of the Bruce test for COVID-19 survivors. For the first stage (S1), significant differences were found among the groups for SpO_2_ (F_2,165_ = 18.92; *p* < 0.001; *ƞ* ^*2*^ = 0.19 - *large*), with higher values in non-hospitalized group when compared to hospital (*p =* 0.004; *d =* 0.61 - *moderate*) and ICU (*p <* 0.001; *d =* 1.17—*large*), and higher values were observed in hospital than ICU group (*p =* 0.010; *d =* 0.57—*moderate*). Also, a significant difference was identified for RQ (F_2,163_ = 14.71; *p* < 0.001; *ƞ* ^*2*^ = 0.15—*large*), with higher values in non-hospitalized group when compared to hospital (*p <* 0.001; *d =* 0.99—*large*) and ICU (*p =* 0.001; *d* = 0.70—*moderate*) groups. No significant differences were detected for HR, VE, VO_2_peak, VO_2_/HR, and RPE (*p >* 0.05). 100% of the patients performed the S1 stage.

**TABLE 2:** Physiological variables in each stage of the Bruce test for COVID-19 survivors.

Stage	HR (bpm)			VE (L/min)			VO_2_ peak (ml.kg^−1^.min^−1^)			VO_2_/HR		
	NH	Hospital	ICU	NH	Hospital	ICU	NH	Hospital	ICU	NH	Hospital	ICU
S1	121.2 ± 20.42	119.8 ± 22.9	124.73 ± 21.6	23.9 ± 8.0	23.9 ± 8.3	25.9 ± 9.4	13.9 ± 3.7	14.6 ± 4.6	14.7 ± 4.3	10.6 ± 4.0	11.4 ± 4.2	10.6 ± 3.3
S2	135.2 ± 18.9	135.9 ± 23.3	137.7 ± 22.6	34.0 ± 10.9	33.5 ± 11.8	34.5 ± 11.8	18.3 ± 4.5	18.6 ± 5.1	17.9 ± 4.6	12.5 ± 4.2	12.6 ± 4.2	11.9 ± 3.4
S3	155.8 ± 16.9	151.7 ± 23.5	149.0 ± 22.3	48.8 ± 16.8	42.7 ± 15.9	45.5 ± 19.0	23.0 ± 6.3	21.6 ± 6.3	21.2 ± 6.4	13.6 ± 5.1	13.3 ± 4.4	13.0 ± 4.2
S4	172.5 ± 15.6**	155.7 ± 22.1	155.6 ± 21.5	66.3 ± 21.7**	52.9 ± 21.9	49.2 ± 14.5	28.3 ± 7.6‡	25.4 ± 9.1	22.8 ± 5.4	14.7 ± 5.1	15.1 ± 4.7	13.5 ± 4.1
S5	172.9 ± 20.7	165.3 ± 16.0	151.8 ± 20.1	61.5 ± 25.3	55.6 ± 17.7	57.6 ± 10.6	29.6 ± 9.9	26.9 ± 9.8	25.3 ± 3.8	14.0 ± 6.6	17.2 ± 4.4	17.8 ± 3.2
S6^a^	185.0 ± 4.24	174.5 ± 0.7	163.0	98.0 ± 5.3	68.9 ± 13.4	72.5	33.7 ± 7.0	29.2 ± 0.3	25.5	14.8 ± 0.4	17.7 ± 5.6	19.2

Note: Numerical data is described as mean and standard deviation (SD). Categorical data is described as absolute and relative (%) frequencies. NH, non-hospitalized; ICU, intensive care unit; S1 = Stage 1; S2 = Stage 2; S3 = Stage 3; S4 = Stage 4; S5 = Stage 5; S6 = Stage 6; HR, heart rate; VE, ventilation; VO_2_peak = peak oxygen consumption; VO_2_/HR, relationship between peak oxygen consumption and heart rate. * = Statistically significant difference between NH, and ICU, groups (*p* < 0.05); ** = Statistically significant difference between NA, and Hospital/ICU, groups (*p* < 0.05); ‡ = Statistically significant difference between NH, and Hospital groups (*p* < 0.05); † = Statistically significant difference between Hospital and ICU, groups (*p* < 0.05); ^a=^ICU, group values have no SD, reported since only one individual from this group performed S6.

**TABLE 3:** Physiological, subjective variables and frequencies of participants in each stage of the Bruce Test for COVID-19 survivors.

Stage	RQ			RPE (a.u.)			min.SpO_2_ (%)			n (%)		
	NH	Hospital	ICU	NH	Hospital	ICU	NH	Hospital	ICU	NH	Hospital	ICU
S1	1.2 ± 0.3**	1.0 ± 0.1	1.0 ± 0.2	8.9 ± 2.3	9.7 ± 3.2	9.5 ± 2.9	97.1 ± 1.4**	96.0 ± 2.0†	94.9 ± 2.2	61 (100.0)	58 (100.0)	52 (100.0)
S2	1.1 ± 0.1**	1.0 ± 0.1	1.0 ± 0.1	11.5 ± 3.4	12.3 ± 3.5	13.1 ± 3.6	96.8 ± 1.5*	95.7 ± 2.3†	94.0 ± 3.2	59 (96.7)	57 (98.3)	51 (98.1)
S3	1.2 ± 0.2**	1.0 ± 0.1	1.1 ± 0.1	14.2 ± 3.5	15.2 ± 4.0	15.3 ± 4.3	96.5 ± 1.9*	95.5 ± 2.6†	93.5 ± 3.2	56 (91.8)	51 (87.9)	41 (78.8)
S4	1.3 ± 0.2**	1.1 ± 0.1	1.1 ± 0.1	16.0 ± 3.1	15.8 ± 3.7	15.5 ± 4.9	96.1 ± 2.0*	94.9 ± 2.0†	92.3 ± 4.1	35 (57.4)	29 (50.0)	21 (40.4)
S5	1.4 ± 0.2	1.1 ± 0.0	1.1 ± 0.1	16.4 ± 2.1	14.8 ± 2.3	11.4 ± 3.0	95.1 ± 3.2	93.9 ± 3.5	91.0 ± 7.0	17 (27.9)	8 (13.8)	5 (9.6)
S6^a^	1.8 ± 0.4	1.1 ± 0.0	1.0	19.0 ± 1.4	15.5 ± 3.5	18.0	89.5 ± 2.1	95.0 ± 2.8	93.0	2 (3.3)	2 (3.4)	1 (1.9)

Note: Numerical data is described as mean and standard deviation (SD). Categorical data is described as absolute and relative (%) frequencies. NH, non-hospitalized; ICU, intensive care unit; S1 = Stage 1; S2 = Stage 2; S3 = Stage 3; S4 = Stage 4; S5 = Stage 5; S6 = Stage 6; RQ, respiratory quotient; RPE, rating of perceived exertion; SpO_2_ = minimum peripheral oxygen saturation; n = number of participants. * = Statistically significant difference between NH, and ICU, groups (*p* < 0.05); ** = Statistically significant difference between NA, and Hospital/ICU, groups (*p* < 0.05); ‡ = Statistically significant difference between NH, and Hospital groups (*p* < 0.05); † = Statistically significant difference between Hospital and ICU, groups (*p* < 0.05); ^a=^ICU, group values have no SD, reported since only one individual from this group performed S6.

For the second stage (S2), significant differences were found between the groups for SpO_2_ (F_2,162_ = 18.34; *p* < 0.001; *ƞ* ^*2*^ = 0.18 - *large*), with higher values in non-hospitalized group when compared to ICU group (*p <* 0.001; *d =* 1.16—*large*), and higher values were identified for hospital when compared to ICU group (*p <* 0.001; *d =* 0.72—*moderate*). Besides, a significant difference was identified for RQ (F_2,163_ = 15.09; *p* < 0.001; *ƞ* ^*2*^ = 0.16—*large*), with higher values in non-hospitalized group when compared to hospital (*p <* 0.001; *d =* 0.98—*large*) and ICU (*p =* 0.001; *d* = 0.74—*moderate*) groups. No significant differences were observed for HR, VE, VO_2_peak, VO_2_/HR, and RPE (*p >* 0.05). 59 (96.7%) of non-hospitalized, 57 (98.3%), hospitalized and 51 (98.1%) of ICU patients completed this stage (S2).

For the third stage (S3), significant differences were found between the groups for SpO_2_ (F_2,141_ = 15.63; *p* < 0.001; *ƞ* ^*2*^ = 0.18—*large*), with higher values in non-hospitalized group when compared ICU group (*p <* 0.001; *d =* 1.16—*large*)*,* and higher values were identified for hospital when compared to ICU group (*p =* 0.001; *d =* 0.77—*moderate*). Moreover, a significant difference was detected for RQ (F_2,144_ = 23.96; *p* < 0.001; *ƞ* ^*2*^ = 0.25—*large*), with higher values for in non-hospitalized group when compared to hospital (*p <* 0.001; *d* = 1.24—*large*) and ICU (*p =* 0.001; *d* = 1.10—*large*) groups. No significant differences were observed for HR, VE, VO_2_peak, VO_2_/HR, and RPE (*p >* 0.05). 56 (91.8%) of non-hospitalized, 51 (87.9%), hospitalized and 41 (78.8%) of ICU patients completed this stage (S3).

For the fourth stage (S4), significant differences were observed for HR (F_2,82_ = 7.73; *p* < 0.001; *ƞ* ^*2*^ = 0.16—*large*), with higher values in non-hospitalized group when compared to hospital (*p =* 0.003; *d =* 0.87—*large*) and ICU (*p =* 0.006; *d =* 0.87—*large*) groups. Significant differences were detected for VE (F_2,81_ = 5.71; *p* = 0.005; *ƞ* ^*2*^ = 0.12—*moderate*), with higher values in non-hospitalized group when compared to hospital (*p* = 0.028; *d =* 0.66—*moderate*) and ICU (*p* = 0.009; *d =* 0.84—*large*) groups. Besides, significant differences were detected for VO_2_peak (F_2,82_ = 3.48; *p* = 0.036; *ƞ* ^*2*^ = 0.08—moderate), with higher values in non-hospitalized group when compared to ICU group (*p =* 0.03; *d =* 0.72—*moderate*). Significant differences were observed for RQ (F_2,81_ = 30.86; *p* < 0.001; *ƞ* ^*2*^ = 0.43—large), with higher values in non-hospitalized group when compared to hospital (*p <* 0.001; *d* = 1.82—*large*) and ICU (*p <* 0.001; *d* = 1.62—*large*) groups. Also, significant differences were observed for SpO_2_ (F_2,78_ = 13.37; *p* < 0.001; *ƞ* ^*2*^ = 0.26—*large*), with higher values in non-hospitalized group than compared to ICU group (*p <* 0.001; *d =* 1.43—*large*), and higher values in hospital group when compared to ICU group (*p =* 0.003; *d =* 0.08—*small*). No significant differences were detected for VO_2_/HR and RPE (*p >* 0.05). 35 (57.4%) of non-hospitalized, 29 (50.0%), hospitalized and 21 (40.4%) of ICU patients completed this stage (S4).

For the fifth stage (S5) and For the sixth stage (S6), was not possible to perform statistical analysis - in order to not to make a type 1 error, since the number of participants in these stages was low [S5: 17 (29.7%) of non-hospitalized, 8 (13.8%), hospitalized and 5 (9.6%) of ICU patients completed this stage, and S6: 2 (3.3%) of non-hospitalized, 2 (3.4%), hospitalized and 1 (1.9%) of ICU patients completed this stage].

**FIGURE 2:**
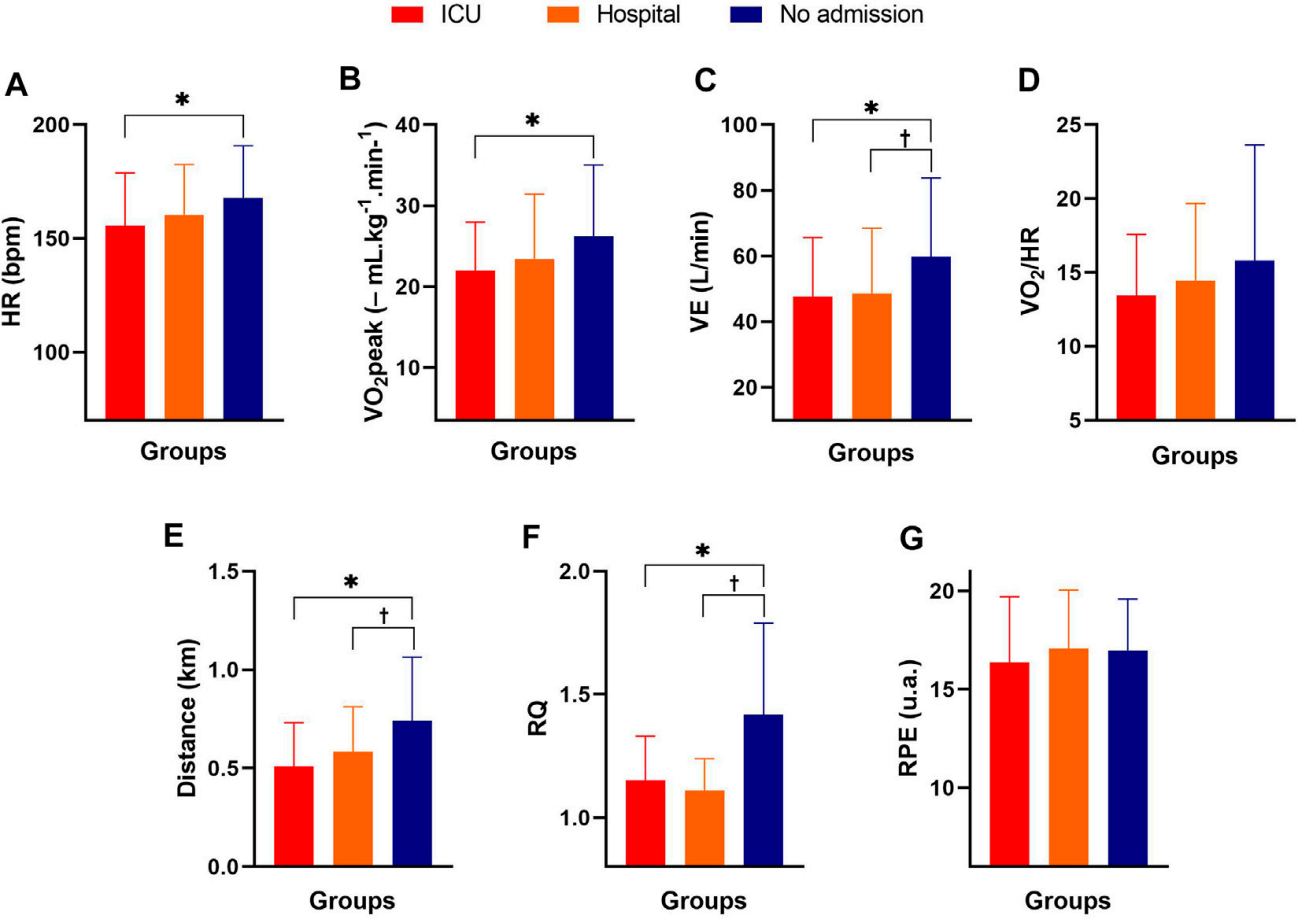
Cardiopulmonary parameters of male and female COVID-19 survivors in the ICU, hospitalized, and non-hospitalized groups. Note: data are expressed as the mean and standard deviation; Panel **(A)** = HR (heart rate); Panel **(B)** = VO_2_ peak (peak oxygen consumption); Panel **(C)** = VE (ventilation); Panel **(D)** = VO_2_/HR (relationship between oxygen consumption and heart rate); Panel **(E)** = distance; Panel **(F)** = VCO_2_/VO_2_ (ratio between respiratory exchanges); Panel **(G)** = RPE (rating of perceived exertion); * = significant difference between the non-hospitalized group and the ICU group; † = significant difference between the non-hospitalized and hospitalized groups. One-way ANOVA with Bonferroni post hoc test. Level of significance established = *p* < 0.05.

Significant differences were observed between the groups for fat mass (F_2,168_ = 3.82; *p =* 0.024; *ƞ* ^*2*^ = 0.04—*small*) and body fat percentage (F_2,168_ = 3.55; *p =* 0.031; *ƞ* ^*2*^ = 0.04—*small*), with higher values in the hospitalized group compared to the non-hospitalized group (*p =* 0.028, *d =* 0.48—*moderate*; *p =* 0.049, *d =* 0.44—*moderate*, respectively). No significant differences were found among the groups for total body mass, height, BMI, lean mass, musculoskeletal mass, or fat-free mass (*p >* 0.05). Figure 2 shows the cardiopulmonary parameters of the men and women who survived COVID-19 in the ICU, hospitalized, and non-hospitalized groups.

**FIGURE 3:**
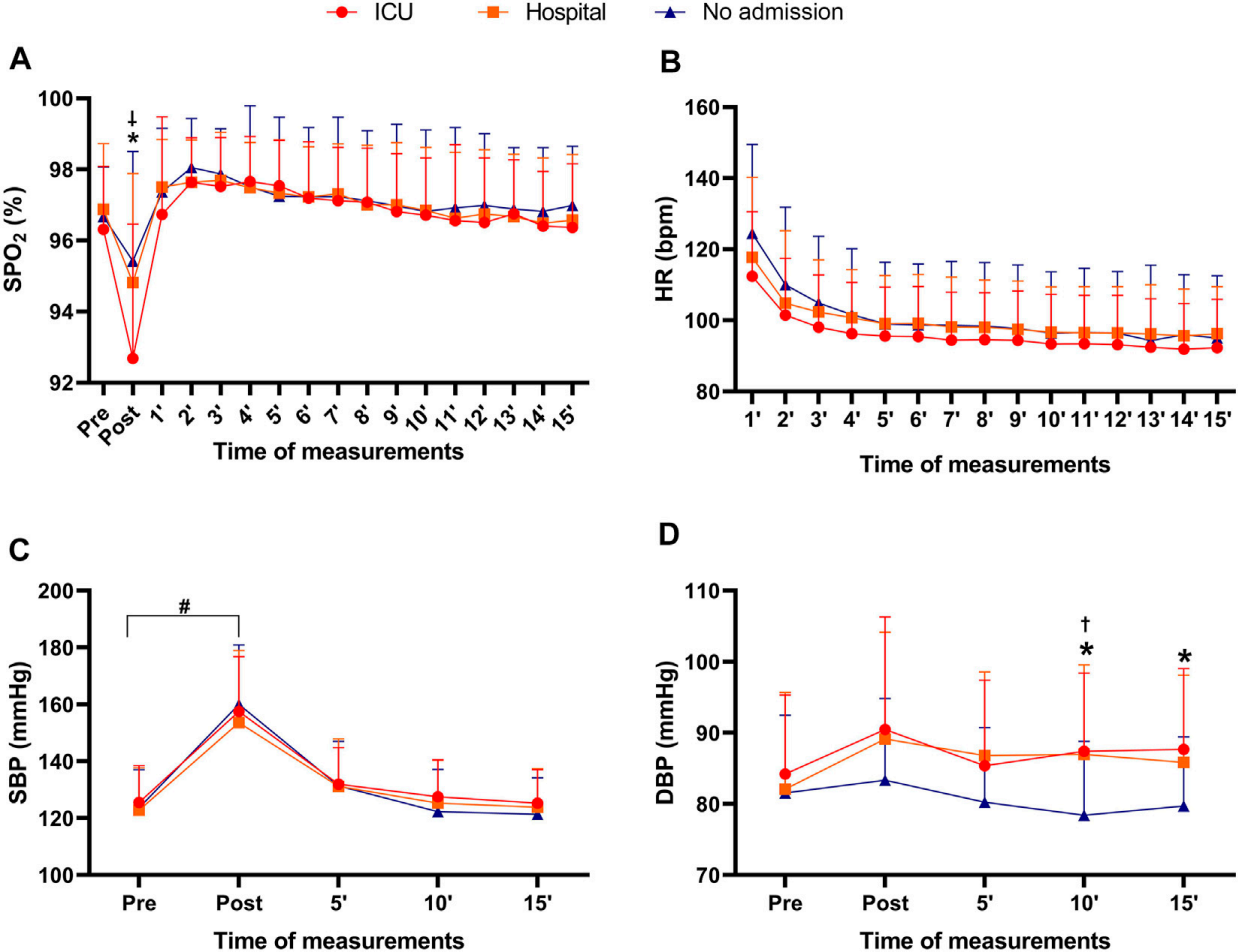
Peripheral oxygen saturation, heart rate, systolic and diastolic blood pressure before, during, and after Bruce testing at different times in COVID-19 survivors in the ICU, hospitalized, and non-hospitalized groups. Note: data are presented as the mean and (±) standard deviation; Panel **(A)** = peripheral oxygen saturation before the Bruce test, subsequently after, and for 15 min after the test; Panel **(B)** = heart rate at the end of the Bruce test and for 15 min after the test; Panel **(C)** = pre-test systolic blood pressure, subsequently after the test, 5 min after the test, 10 min after the test and 15 min after the test; Panel **(D)** = pre-test diastolic blood pressure, subsequently after the test, 5 min after the test, 10 min after the test and 15 min after the test; %SPO_2_ = peripheral oxygen saturation; HR = heart rate; SBP = systolic blood pressure; DBP = diastolic blood pressure; * = significant difference between non-hospitalized and ICU; † = significant difference between the non-hospitalized and hospitalized; ‡ = significant difference between ICU and hospitalized; # = significant difference between pre and post times for the three experimental groups. Two-way ANOVA with Bonferroni post hoc test. Level of significance established = *p* < 0.05.

Significant differences were found among the groups for HR (F_2,168_ = 4.17; *p =* 0.017; *ƞ* ^*2*^ = 0.05—*small*), with higher values for the non-hospitalized group than for the ICU group (*p =* 0.015; *d =* −0.54—*moderate*). For VE, significant differences were also observed among the groups (F_2,168_ = 6.15; *p =* 0.003; ƞ^2^ = 0.07 - *moderate*), with higher values for the non-hospitalized group compared to the ICU group (*p =* 0.007; *d =* −0.58—*moderate*) and hospital (*p =* 0.012; *d =* −0.54—*moderate*). For VO_2_peak, a significant difference was also observed among the groups (F_2,168_ = 4.41; *p =* 0.014; *ƞ* ^*2*^ = 0.05—*small*), with higher values for the non-hospitalized group than for the ICU group (*p =* 0.013; *d =* −0.55—*moderate*). Significant differences were also identified between the groups for the distance covered in the test (F_2,168_ = 11.02; *p <* 0.001; *ƞ* ^*2*^ *=* 0.12 - *moderate*), with higher values for the non-hospitalized group when compared to the ICU group (*p <* 0.001; *d =* −0.86—*large*) and hospitalized group (*p =* 0.006; d = −0.58—*moderate*). For the RQ, significant differences were observed between the groups (F_2,168_ = 25.65; *p <* 0.0001; *ƞ* ^*2*^ = 0.23—*large*), with higher values for the non-hospitalized group than for the ICU (*p <* 0.0001; d = 0.91—*large*) and hospitalized (*p <* 0.0001; *d =* 1.10—*large*) groups. No significant differences were found between the groups for VO_2_/HR or the RPE post-Bruce test (*p >* 0.05). Figure 3 shows the %SPO_2_, HR, SBP, and DBP behavior before, during, and after the Bruce test at different measurement times in the three groups.

For the post Bruce test %SpO_2_, significant differences were identified among the groups (F_2,165_ = 10.12; *p <* 0.001; *ƞ* ^*2*^ = 0.11—*moderate*), with higher values for the non-hospitalized group when compared to the ICU group (*p <* 0.0001; *d =* 0.79—*moderate*) and for the hospitalized group when compared to the ICU group (*p <* 0.0001; *d =* 0.62—*moderate*); there were no other significant differences (*p >* 0.05). Additionally, it was observed that the %SpO_2_ of the ICU (*p <* 0.0001; *d =* 1.22—*large*) and hospitalized (*p <* 0.0001; *d =* 0.08—*small*) post Bruce test groups showed lower values when compared to the pre-test time. For HR, no significant differences were observed among the groups during the 15 min after the Bruce test (*p >* 0.05). For SBP, higher values were found post-test when compared to pretest for the ICU (*p <* 0.0001; *d =* 1.94—*large*), hospitalized (*p <* 0.0001; *d =* 1.48—*large*) and non-hospitalized groups (*p <* 0.0001; *d =* 2.08—*large*). No significant differences were observed for SBP in subsequent measurements in the three experimental groups (*p >* 0.05). For DBP, significant differences were observed (Bruce’s post-test) (F_2,168_ = 4.17; *p =* 0.017; *ƞ* ^*2*^ *=* 0.05—*moderate*), with higher values for the ICU group than for the non-hospitalized group (*p =* 0.025; *d =* 0.29—*moderate*). Furthermore, the DBP of the non-hospitalized group 10 minutes after the exercise test was significantly lower than the values presented by the ICU (*p <* 0.008; *d =* 0.83—large) and hospitalized groups (*p <* 0.01; *d =* 0.74—*moderate*) at the same measurement point. Finally, 15 minutes after the Bruce test, the blood pressure of the non-hospitalized group remained lower than the DBP of the ICU group (*p <* 0.04; *d =* 0.75—*moderate*). The three experimental groups identified no significant differences between pre-test %SpO_2_, pre-test SBP, pre-test DBP, or final SBP (*p >* 0.05).

## Discussion

The main results of this study indicated 1) a higher level of self-reported physical activity for the non-hospitalized group when compared to the hospital and ICU groups; 2) fat mass and body fat percentage were significantly higher in the hospitalized group when compared to the non-hospitalized group; 3) the final HR and VO_2_peak after the Bruce test was significantly higher in the non-hospitalized group when compared to the ICU group; 4) the distance covered in the Bruce test was significantly greater in the non-hospitalized group when compared to the hospitalized and ICU groups; 5) %SPO_2_ was lower after the Bruce test in the ICU group when compared to the non-hospitalized group; and 6) the final post-Bruce test DBP was significantly higher in the ICU and hospitalized groups.

On the other hand, no significant differences were identified among the three experimental groups for comorbidities associated with obesity, smoking, lean mass, musculoskeletal mass, fat-free mass, VO_2_/HR, RPE, or HR after the Bruce test over 15 min of measurement. The study hypothesis was confirmed, considering the differences in body composition and cardiorespiratory fitness in non-hospitalized and hospitalized individuals.

There was no significant difference in the body mass index among the different groups, i.e., ICU, hospitalized, and non-hospitalized, although there was a significant difference in body fat percentage, with lower values for the non-hospitalized group than the hospitalized group. Thus, it is conjectured that not just BMI but the higher body fat percentage may contribute to influencing the outcome of COVID-19. Excess body fat promotes hyper inflammation at a systemic level, with the secretion of proinflammatory mediators, such as cytokines, adipokines, and chemokines, with a consequent reduction in the immune response (Maffetone and Laursen, 2020). It is possible to affirm that being overweight and obese promoted a significant worsening of the symptoms of COVID-19, but prior assessments were not carried out before the pandemic.

However, actions that encourage the regular and systematic practice of physical activity, i.e., with emphasis on resistance exercises to promote changes in body composition, with a reduction in fat mass and an increase in musculoskeletal mass (with a consequent change in body fat percentage, in addition to aerobic exercise, to improve cardiorespiratory conditioning (Garber et al., 2011), have become essential for improving physical fitness and thus the health of the population. The literature has already pointed out an inverse correlation between the level of physical activity and accumulated deaths from COVID-19 (Pitanga et al., 2021).

The cardiorespiratory fitness of the hospitalized and ICU groups was significantly lower than that of the non-hospitalized group. Cardiorespiratory assessment mainly aims to verify the patients’ physical capacity, effort tolerance, and possible cardiopulmonary abnormalities (Fletcher et al., 2013). Silva et al. (2021) found that hospitalization rates for COVID-19 among endurance athletes were significantly lower than expected. Brandenburg et al. (2021) pointed out that healthy individuals with better cardiorespiratory fitness (able to walk 4.8 km without feeling extremely tired and able to perform slow and fast walking and running) had a lower hospitalization rate than individuals with lower cardiorespiratory fitness. Because of this, the two pieces of evidence mentioned earlier (Brandenburg et al., 2021; Silva et al., 2021) suggest that cardiorespiratory fitness has a cardioprotective effect, reducing hospitalization rates, like that observed in the present study, i.e., individuals with lower cardiorespiratory fitness had more severe symptoms of COVID-19, although we cannot confirm such differences, given the experimental design of the present study. Besides that, it was essential to emphasize that low levels of cardiorespiratory fitness could impact obese adolescents (Alemayehu et al., 2018; Salvadego et al., 2018). Thus, public policies to promote physical activity, healthy nutrition and safe mental health are indispensable in the early stages of life (Branco et al., 2019; Branco et al., 2020; Branco et al., 2021).

Additionally, Sallis et al. (2021) suggested that regular and systematic physical activity following established guidelines of 150 min/week reduced hospitalization rates to 3.2% in the hospital, 1% in the intensive care unit, and 0.4% for deaths among 3,118 patients who had COVID-19. Therefore, it can be inferred that the hospitalization rate, both in the ward and in the intensive care unit, is higher among individuals with low cardiorespiratory fitness and with a lower level of physical activity when compared to individuals with higher cardiorespiratory fitness and a higher level of physical fitness (Af Geijerstam et al., 2021; Christensen et al., 2021). The present study indicated a significant difference in physical activity among the groups, i.e., the non-hospitalized group showed higher physical activity levels than both hospitalized groups (before COVID-19 contraction). In Brazil, between January and July of 2021, several places, including gyms, squares, and parks, in addition to other places that were possible to practice physical activity, were closed due to COVID-19. Many decrees authorized and disallowed access to public places. Thus, the physical activity level declined a lot during this period. Given this, it is not possible to establish a relationship between cause and effect between physical activity and COVID-19, although the level of physical activity values was higher in the non-hospitalized group when compared to the hospitalized ones.

VE during the Bruce test was higher in the non-hospitalized group than in the hospitalized and ICU groups. The increased intensity can explain these differences in the test differed between the participants (Herdy et al., 2016). Concerning VO_2_peak, Barbagelata et al. (2021) found significantly lower values for patients with post-COVID-19 syndrome than for asymptomatic subjects (25.8 ± 8.1 ml kg^−1^. min^−1^ vs. 28.8 ± 9.6 ml kg^−1^. min^−1^; *p =* 0.017). These responses may be related to the long period of physical inactivity that resulted in cardiorespiratory deconditioning, residual inflammation (convalescent phase), possible systemic and/or organ damage, prolonged invasive or noninvasive ventilation, poor health conditions, or even the sum of the conditions (Carfi and Bernabei, 2020). In addition, long-term functional impairment after hospital discharge for COVID-19, particularly for those admitted to the ICU, stands out as a possible sequela (Clavario et al., 2021). Consequently, the reduced performance on the cardiorespiratory fitness test of the hospitalized and ICU groups would be justified by the deleterious effects of the sum of the symptoms of COVID-19, i.e., a lower VO_2_ peak and shorter distance covered in the test.

It is also worth mentioning that the RPE did not present a significant difference among the groups, indicating that the perceived intensity was similar. Furthermore, it was found that the distance covered during the Bruce test was significantly longer in the non-hospitalized group than in the two hospitalized groups. The worse performance on the Bruce test of patients in the hospitalized groups can be explained by possible sequelae of COVID-19 related to myositis and myalgia (associated with the severity of symptoms of COVID-19) (Paliwal et al., 2020), physical deconditioning promoted by hospitalization and a lack of neuromuscular stimuli (Solverson et al., 2016), and a reduced cardiorespiratory capacity (Af Geijerstam et al., 2021; Brandenburg et al., 2021; Silva et al., 2021). For this reason, carrying out health-related physical fitness tests (tests to measure muscle strength, muscle endurance, flexibility, and cardiorespiratory conditioning, in addition to assessing body composition to verify lean mass, fat, and body fat percentage) are essential to more assertively direct rehabilitation/training and nutrition programs.

Physical inactivity is not solely responsible for the worsening symptoms of COVID-19. Petrovic et al. (2020) reported that the cytokine storm resulted from an increase in low-grade inflammation due to obesity and associated comorbidities (Maffetone and Laursen, 2020), and even the use of immunosuppressants was related to the worsening symptoms of COVID-19, additionally to heart disease, dysregulation of the renin-angiotensin-aldosterone system, plaque destabilization (causing acute coronary syndrome), and the promotion of a prothrombotic state and clotting disorders (Silva et al., 2022). In congruence with the American College of Sports Medicine (Pescatello, 2005), DBP elevation is not typical during a cardiorespiratory test. Potential mechanisms associated with the hypertensive response of post-Bruce DBP can be explained by the excessive elevation of the double product that can result in global subendocardial ischemia due to an inability to maintain myocardial oxygen supply and demand (Ha et al., 2002). On the other hand, Sydó et al. (2018) point out that the etiology of an increase in DBP after physical exercise is not fully elucidated in the literature; possible risk factors or cardiovascular disease may not be associated with increased DBP. The same authors suggest that the central focus of analysis should be on cardiorespiratory capacity and recovery heart rate, although the present study did not identify a difference in HR during the 15 min of measurement. Recently, Freire et al. (2022) pointed out that people with post-COVID-19 obesity had an increase in the stress index and a reduction in parasympathetic activity compared to people without obesity who were discharged after COVID-19. However, the present study did not measure heart rate variability in the fast and slow recovery phases after physical effort, which can be considered a limitation. Another limitation of this study was a lack of control of respiratory frequency after the Bruce test; thus, transposing to practice, respiratory rate monitoring before, during and post-exercise could be investigated in post-COVID-19 patients with different symptoms.

Another point that deserves attention is related to post-COVID-19 systemic arterial hypertension. Chen et al. (2021) pointed out that 93% of critically ill patients (hospitalized in an intensive care unit) had cardiac lesions, and systemic arterial hypertension may be a sequela of COVID-19. Therefore, troponin I and angiotensin-2 monitoring can monitor hemodynamic parameters in serial assessments, as Chen et al. (2021) described. Considering that the responses of the cardiopulmonary system can be dysfunctional, the practice of recreational or high-performance physical activity should be carefully evaluated by a multidisciplinary team (Colombo et al., 2021). The type, volume, and intensity of physical exercise should be analyzed and monitored before, during, and after the sessions via %SpO_2_, HR, respiratory frequency, and blood pressure and recorded to compare with the subsequent sessions to analyze the impact of the physiological stress caused by exercise rehabilitation/adaptation on post-COVID-19 patients.

Post-COVID-19 patients will need post-hospital care to minimize possible biopsychosocial sequelae so that the patient does not become “invisible” to society. However, early mobilization strategies can be adopted to reduce possible sequelae resulting from COVID-19, following the guidelines for early mobilization in an intensive care unit (Aquim et al., 2019). An additional possible limitation of this study is the absence of measurement of heart rate variability to monitor the rapid and slow rate phases of heart rate variability, as well as to the study design, i.e., being cross-sectional, which does not allow for a cause and effect relationship to be delineated. As a strong point, morphological and cardiorespiratory aspects that need rehabilitation and are linked to physical fitness related to health were verified. Therefore, strategies for recovering from health conditions through physical activity and incorporating a healthy diet become essential for COVID-19 survivors, especially those who are symptomatic. Public policies for stimulating physical activity, healthy nutrition, and reducing tobacco are indispensable independently of COVID-19. Finally, it is suggested to periodically monitor body composition and cardiorespiratory variables to verify possible sequelae related to COVID-19 and organic behavior in the face of physical stress.

## Conclusion

Based on the present study’s findings, it is concluded that fat mass and body fat percentage were significantly higher in hospitalized post-COVID-19 participants. The cardiorespiratory fitness of the hospitalized and ICU groups was significantly lower than the non-hospitalized group, especially the ICU group, although there was no significant difference among the groups for RPE in the post-Bruce test. Vital signs were significantly different in hospitalized participants compared to non-hospitalized participants (after the Bruce test: lower %SPO_2_ and higher DBP in hospitalized participants), suggesting specific actions based on responses to rehabilitate the survivors (especially those who were hospitalized). Another point that should be considered is the vital signs before, during, and after cardiopulmonary rehabilitation; the multidisciplinary team must monitor HR, SpO_2,_ and blood pressure are necessary during rehabilitation to avoid possible physical complications. The volume and intensity of physical exercises should be adjusted, conforming to the physiological adaptation of the patients. Although the experimental design does not allow the relationship between a healthy lifestyle and cause and effect, the scientific literature already points out pieces of information more than necessary to improve physical activity, promote healthy nutrition, and reduce tobacco to improve health. Thus, behavioral changes and public policies are indispensable to promoting health and reducing hospitalization costs. Finally, it is considered essential and urgent to improve the body composition and cardiorespiratory fitness of overweight and obese COVID-19 survivors independently of hospitalization.

## Data Availability

The raw data supporting the conclusion of this article will be made available by the authors, without undue reservation.
